# Poly-Lactic Acid-Bagasse Based Bio-Composite for Additive Manufacturing

**DOI:** 10.3390/polym15214323

**Published:** 2023-11-04

**Authors:** Silvia Carichino, Dino Scanferla, Daniela Fico, Daniela Rizzo, Francesca Ferrari, María Jordá-Reolid, Asunción Martínez-García, Carola Esposito Corcione

**Affiliations:** 1 Department of Engineering for Innovation, University of Salento, Edificio P, Campus Ecotekne, s.p. 6 Lecce-Monteroni, 73100 Lecce, Italy; silvia.carichino@unisalento.it (S.C.); scanferladino@gmail.com (D.S.); daniela.fico@unisalento.it (D.F.); francesca.ferrari@unisalento.it (F.F.); 2 Department of Cultural Heritage, University of Salento, via D. Birago 64, 73100 Lecce, Italy; daniela.rizzo@unisalento.it; 3 AIJU, Technological Institute for Children’s Products and Leisure, Ibi, 03440 Alicante, Spain; mariajorda@aiju.es (M.J.-R.); sunymartinez@aiju.es (A.M.-G.)

**Keywords:** bagasse, poly-lactic acid (PLA), bio-composites, additive manufacturing, 3D printing

## Abstract

Beer bagasse is a residue waste produced in great amounts; nevertheless, it is still underestimated in the industry. The aim of this paper is to develop an innovative and efficient methodology to recycle the beer bagasse by producing Poly-lactic acid(PLA)-based bio-composites, in the forms of pellets and filaments, to be used in additive manufacturing processes. To assess the suitability of beer bagasse for extrusion-based 3D printing techniques, it was, firstly, physically and chemically characterized. Then, it was added in combination with different kinds of plasticizers to PLA to make bio-composites, analyzing their thermal and physical properties. The results prove the great potential of bagasse, evidencing its printability. Both composites’ pellets and filaments were used in two different 3D printing machines and the mechanical properties of the 3D-printed models were evaluated as a function of the composition and the kind of technology used. All the used plasticizers improved processability and the polymer–bagasse interface. Compared to neat PLA, no changes in thermal properties were detected, but a lowering of the mechanical properties of the 3D-printed composites compared to the neat polymers was observed. Finally, a comparison between the efficiency of the two 3D printing techniques to be used with the bio-based composites was performed.

## 1. Introduction

Beer is one of the most commonly drank beverages and a growth in customer demand implies an ongoing growth in its production. The intensification of the production of beer causes a consequent increase in the fixed wastes generated during its production. The most abundant beer waste is bagasse (spent brewers’ grain), rising from the filtering and pressing of the malt obtained after the saccharification of the malted barley grain [1]. Its composition is extremely variable, mostly due to the production process, neat materials, and the industrial advancement of the sector [1,2]. Several studies evaluate the possibility to re-use beer bagasse, for the recovery of energy [1,3], the production of xylanase and cellulase [1,4] or bioplastics [1,5]. To the best of our knowledge, no studies reported the possibility to re-use beer bagasse to produce bio-composites to be used in additive manufacturing techniques, but for other processes [6]. Additive manufacturing (AM), also known as three-dimensional (3D) printing, according to standard [7], refers to those technologies that, based on a geometrical representation from 3D model data, create parts by successive addition of material layer-upon-layer [8,9,10]. This latter approach allows the fabrication of complex geometries, with low wastage production [11,12]. For this reason, AM techniques can be considered suitable technologies to reach one of the most important and current scientific and social aims, i.e., the transition from the linear economy model to a circular model. Some of the authors of this paper have already demonstrated that the correct use of AM can have a strategic impact on the economy and on environmental pollution, not only reducing waste production, but also obtaining new products with high added value, driving the industry towards economically and environmentally sustainability [10,13,14,15,16,17,18,19,20]. In this framework, this paper is aimed at studying the possibility to add beer bagasse to PLA, in presence of different kinds of natural plasticizers, to produce bio-composites in the forms of pellets and filaments. Bio-composites are composite materials composed of biodegradable natural fibers or fillers as reinforcement and biodegradable (or non-biodegradable) polymers as the matrix [15]. In particular, natural and recycled fillers can be used to produce fully biodegradable green composites, representing an innovative and efficient solution for current environmental issues. Among the advantages of bio-composites, in fact, the most important are the low energy and low CO_2_ emissions associated with their processing, biodegradability, renewability, low specific weight, higher specific strength, stiffness, high electrical resistance, low cost, and good thermal and acoustic insulating properties. However, most of them present some disadvantages, such as high sensitivity to moisture, low durability, and low adhesion between matrix and fiber [15]. Although some of these disadvantages make them unsuitable for certain applications where the specific requirements are too stringent, they may find a use in other applications where severe features are not required. In the literature, several attempts to create bio-composites by recovering wastes and using it as filler are reported: Toro et al. [21] studied the introduction of egg shell in a polypropylene matrix, Li et al. [22] used waste shellfish shell as filler with polypropylene matrix again [23], durian skin waste was employed by Manshor et al. [24] as filler in PLA bio-composites after a pre-treatment, Sing et al. [25] prepared bio-composites made by PLA with wood waste and rise hush, and Lima et al. recovered mango seed waste and used it as reinforcement in PLA matrix [26]. All these works show that it is possible to recover and valorize waste that can be used in the production of bio-composites.

Among 3D printing techniques that can use bio-composites, the material extrusion process (MEX) can use thermoplastic polymers or composites, in the form of filament, dispensed through a nozzle or orifice. Several studies considered PLA-based bio-composites to be printed using MEX techniques: Hong et al. [27] realized a filament of PLA reinforced by modified lignin to be used as feed material in a fused deposition modeling process, Flores-Hernandez et al. [28] showed how to prepare bio-composites of PLA reinforced with rachis obtained from chicken feathers and used it in AM, and Le Duigou et al. employed bio-composites with PLA and continuous flax fibers for structural application [29]. In previous works, PLA filaments for MEX processes with filler derived from scraps of olive wood [16], ceramics [17], and stones [13] were also prepared. Nowadays, new 3D printing machines have substituted the filament extruder with a pellet extruder, thus reducing the cost of the printing process, printing a wider range of materials, and increasing the speed of prints and the chance of recyclability when compared to filament extruder. Overall, this paper reports a comparison between the properties of the bio-composites containing PLA and beer bagasse in the presence of different kinds of compatibilizer, in the form of pellets and filaments. It also evaluates the suitability of both kinds of produced bio-composites in relation to their use for the two selected MEX techniques. Finally, it presents a comparison between the physical–mechanical properties of the 3D-printed model, by using pellets and filaments, in order to evaluate if the feed material could affect the printing quality or the mechanical properties of the formulations. Specifically, we expected the use of pellets to result in better performing objects, considering the facts that they have undergone fewer processing steps.

## 2. Materials and Methods

### 2.1. Materials

In this work, a comparison between the properties of 3D bio-composite samples printed by using two different AM techniques was carried out. To this aim, two extrusion-based 3D printers that required filament and pellets as feed material were selected.

A heat-resistant and high-impact strength grade poly lactic acid (PLA 3D870), supplied by Ingeo (Plymouth, MN, USA, EEUU), was used as a matrix to produce a composite material for 3D printing. The main properties of PLA 3D870, according to the technical data sheet, are reported in Table 1.

A total of 10 wt. % of beer bagasse, a by-product of beer production, supplied by Cervezas Lluna (Alcoy, Spain) was added to PLA, to produce both composite filaments and pellets. 

Several plasticizers have been also added to PLA with a compatibility function: maleic anhydride (MA), provided by Scharlab (Sentmenat, Spain), and epoxidized linseed oil (ELO) and epoxidized soybean oil (ESBO) supplied by Sigma Aldrich (St. Louis, MI, USA). Table 2, Table 3 and Table 4 report the specifications of the three substances.

### 2.2. Bagasse’s Characterization

Bagasse (BG) was firstly ground using rotor mill Retsch ZM300 (Retsch GmbH, Haan, Germania [38] to obtain a powder with a granulometry of 0.5 mm. 

Fourier-transform infrared spectroscopy (FT-IR 6300 Jasco from Jasco Europe s.r.l.) (Cremella, Italy) [39] was carried out on grounded BG samples, spectra were recorded in the 400–4000 cm^−1^ range, using 128 scans and 4 cm^−1^ of resolution by using a germanium round crystal window to determine the characteristic chemical functional groups.

XRD analysis (Rigaku Ultima +, Tokyo, Japan) [40] was carried out on the BG powder with CuKα radiation (λ = 1.5418 Å) in the step-scanning mode recorded in the 2θ range of 10°–60°, with a step size of 0.02° and step duration of 0.5 s. X-ray diffraction is one of the most important non-destructive tools to analyze matter ranging from fluids, to powders and crystals, and it is useful to understand the crystalline or amorphous organization of materials.

Finally, thermal stability of the powder was investigated through thermogravimetric analysis (TA Instruments TGA Q500) (TA instrumens, New Castle, DE 19720, USA) [41], scanning from 0 °C to 565 °C in a N_2_ atmosphere and from 565 °C to 1000 °C in a O_2_ atmosphere, with a heating rate of 10 °C/min. 

### 2.3. Mixture’s Preparation

The bagasse was firstly dried at 40 °C for 72 h in a dryer to remove about 80 wt% of absorbed water, to prevent the appearance of microorganisms and the bagasse from rotting. The temperature was not raised above 40 °C to avoid damaging polyphenols and proteins. 

Then, humidity content was measured using the Cobos FD-720 analyzer (Cobos Precision, Barcelona, Spain) [42], in automatic mode with a measurement deviation of 0.01% and a temperature of 80 °C. The result was 2.86 wt% humidity. In detail, for the measurement of humidity, the method carried out consisted of performing consecutive weighing of the sample, while it was subjected to drying at a constant temperature (80 °C) using IR light lamps. When two consecutive weighings have a weight difference of less than 0.01%, the weight variation is considered null and, therefore, there is no longer a loss of water in the sample. Finally, the equipment takes this last weighing as the final mass of the sample and subtracts it from the initial mass. The percentage of humidity is calculated by dividing the difference between the initial mass and the final mass by the initial mass and multiplying the result by 100, to obtain the result in % of humidity (%h), as shown in the following equation:

(1) $$\%h=\frac{(mf-m0)\times 100}{m0}$$

where *mf* is the weight after drying, *m*0 is the weight before drying. The measurement of the different weights of the sample and the calculation of % humidity is carried out automatically by the measuring equipment.

Before performing the compounding with PLA, both BG and PLA were dried at 80 °C for 4 h to remove traces of moisture in the dryer Arid X10X Dri-air industries (Thompson Rd, East Windsor, NJ, USA) [43] and in a ventilated dryer UF55 Memmert (Schwabach, Germany) [44], respectively. This treatment was necessary to avoid the appearance of microorganisms and fungi, caused by the presence of humidity (between 70 and 85%), lipids, protein matter, and fermentable sugars, responsible for the deterioration of BG properties [1]. Final humidity of BG was 1.05 wt. %.

Three different formulations were prepared by melt compounding of PLA (shaped in pellets), BG, and the compatibilizer using extruder Collin Teach Line ZK25 [45]. The amount of each compatibilizer was 5 wt. % of the mixture PLA/BG (in ratio 90:10). The compositions are shown in Table 5, where each formulation is labelled by specifying the pellet form by “-p”.

Consequently, a filament for each formulation was produced through the extruder Next 1 Advance from 3Devo (Utrecht, The Netherlands) [46], setting a temperature profile for the four extruder zones and the screw speed, as reported in Table 6. The temperature profiles were chosen following extensive research of the literature and optimised for different compositions, considering that the presence of an additive normally causes a viscosity reduction with temperature. In fact, as several works report, the introduction of an additive with a high compatibility with PLA allows for a reduction in glass transition and melting temperatures [47,48,49]. Each formulation in filament form is labelled with “-f”. The PLA–BG formulation could not be extruded because it was too brittle.

### 2.4. Three-Dimensional Printing Techniques

Two different 3D printing techniques were employed. 

NX Pro Pellets (Tumaker (Irun, Spain)) [50] were selected to test the printability of the produced composite pellets, reported in Table 2. 

Witbox 2 from BQ (Madrid, Spain) [51] was used to process the composite filaments of Table 3 as feedstock material.

Tensile specimens were produced by using both 3D printers, to perform characterization tests on the 3D-printed samples and compare the results. The printing parameters used to produce tensile, flexure, and impact specimens, by using both machines, are listed in Table 7, and some of them are explained below:

- The extrusion multiplier determines as a percentage the amount of wire or pellets to be extruded adding or subtracting to the ones calculated. As can be noted, there is a significant difference in this parameter in the two printing techniques. If set above 1, it allows for the creation of more dense prints, but values that are much greater than 1 can sometimes cause material overflow the defines the shape of the printed element, resulting in lower dimensional accuracy but stronger prints [52]. It generally ranges between 0.9 and 1.1, but in the case of Tumaker NX Pro Pellets, it was necessary to set a significantly higher value due to the discontinuity of the feed material;
- The shrink rate represents the polymer shrinkage and it is a form of polymer compensation;
- The first layer width is set to allow a proper adhesion to the substate, and also a speed reduction factor is provided to improve the adhesion.

### 2.5. Characterization Techniques

Glass transition temperature and melting temperature of composite filaments, pellets samples, and 3D-printed models were evaluated by differential scanning calorimetry DSC Q200 calorimeter TA Instruments (New Castle, DE, USA) [53]. Dynamic scans were performed following standard [54], in a N_2_ atmosphere with a flow of 50 mL/min. Each scan consisted of an initial heating cycle from 25 °C to 250 °C at a rate of 10 °C/min and a subsequent cooling down to −90 °C at a rate of 5 °C/min to eliminate eventual stresses. Then, a second scan was performed from 0 °C to 250 °C at a rate of 10 °C/min.

TGA analysis was conducted to investigate thermal stability of the different formulations, according to standard [55] using TA Instruments TGA Q500 [41]. Specifically, a heating from 30 °C to 565 °C in N_2_ atmosphere and a heating from 565 °C to 1000 °C in O_2_ atmosphere were performed, with a gas flow of 50 mL/min. 

Melt flow index (MFI) was also evaluated following standard [56], using Twelvindex Atsfaar [57].

The mechanical behavior of the 3D-printed samples, by using both 3D printing techniques, was examined through tensile, flection, and impact tests. Tensile and flection test occurred using dynamometer Instron 6025 (Canton, MA, USA) [58].

Tensile proves follow the standard [59], at a strain rate of 1 mm/min for the measurement of Young’s modulus and 500 mm/min for the rest of the test. The grips used in these tests were wedge-type grips, the distance between grips was 115 mm, and the load cell was 10 kN. The dimensions of the specimen refer to the 1BA type, according to the standard.

Bending tests were performed according to standard [60] at a strain rate of 2 mm/min. The distance between supports was 64 mm and the load cell used was 10 kN. The specimens tested are straight prism specimens measuring 80 mm × 10 mm × 4 mm. 

Impact tests were carried out using Ceast Resil impactor 6967.000 [61], following the standard [62]. The pendulum used was 5 J and the specimens used were type 1 specimens (prism measuring 80 mm × 10 mm × 4 mm). 

### 2.6. Statistical Analysis

One-way analysis of variance (ANOVA) was used to highlight the statistical significance of the addition of each plasticizer on the mechanical properties. For this purpose, the F value, which is defined as the ratio of the variation between sample means to the variation within the samples, was calculated from the measured data. Then, with “a” being the number of levels of the variance factor and “n” the number of tests for each level, the critical F value, FCV (a − 1, a(n − 1), α), can be estimated. FCV represents the value of F distribution with degrees of freedom (a − 1) and a(n − 1), which, at a confidence level, α, corresponds to the null hypothesis (equivalence of the means). Therefore, F < FCV indicates that the population means are equivalent, whereas F > FCV indicates that the population means are significantly different. Another quantitative measure for reporting the result of a test of hypothesis is the *p*-value. The *p*-value is the probability of the test statistic to be at least as extreme as the one observed, given that the null hypothesis is true. A small *p*-value is an indication that the null hypothesis is false. It is good practice to decide in advance of the test how small a *p*-value is required to reject the test, that is, to choose a significance level, α, for the test. For example, it can be decided to reject the null hypothesis if the test statistic exceeds the critical value (for α = 0.05) or, analogously, to reject the null hypothesis if the *p*-value is smaller than 0.05.

## 3. Results and Discussion

### 3.1. Bagasse’s Characterization

**Figure 1:**
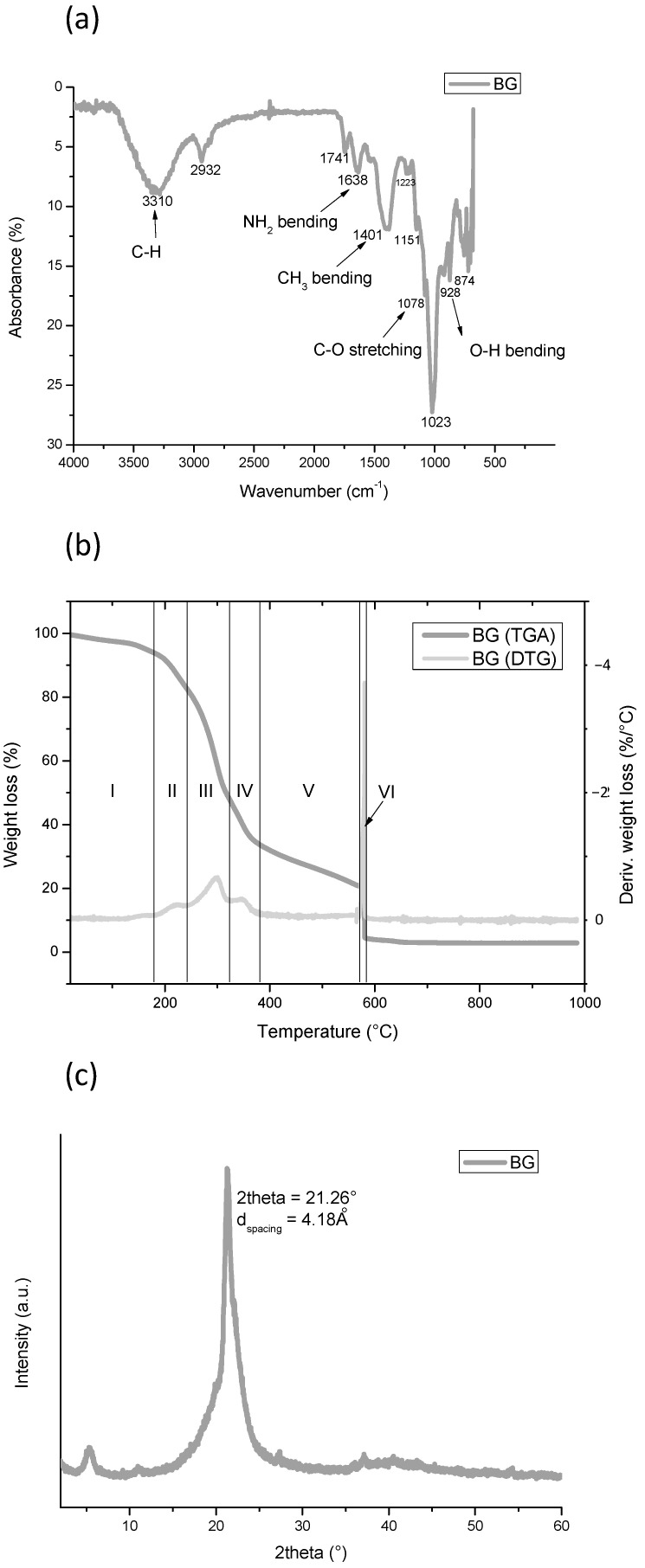
BG characterization: (**a**) FTIR analysis of dried BG; (**b**) TGA and DTG analysis of dried BG; (**c**) XRD analysis of BG.

Bagasse is a typical biomass, containing cellulose, hemicelluloses, and lignin [63]. The chemical characterization of bagasse was carried out using ATR-FTIR spectroscopy: the spectrum is shown in Figure 1a. 

The hydroxyl of cellulose and hemicellulose caused the strong absorbance that happened at the range of 3200–3600 cm^−1^ [63]. The peaks at 2932 cm^−1^ and 2872 cm^−1^ are associated with the asymmetrical and symmetrical stretching vibrations of the C-H groups. The peak at 1741 cm^−1^ corresponds to the C=O stretching of the saturated esters, associated with the hemicellulose present in bagasse. Amino bonds at 1638 cm^−1^, 1521 cm^−1^, and 1401 cm^−1^, which correspond to amide I, amide II, and amide III, respectively, are also evident in the spectrum; these bands are associated with protein, lignin, and polysaccharide biomolecules [1]. Finally, the peaks at 1223 cm^−1^ and 1023 cm^−1^ are associated with the holocellulose biomolecules (cellulose and hemicellulose) [64,65].

As can be seen in Figure 1b, TGA and DTG analysis assessed that the degradation of BG happened in six steps. By observing the two curves, it is possible to identify the start and end temperatures of each step and the temperature at the maximum degradation rate, which can be detected by DTG analysis as the peak temperature of each stage. This experimental result suggests that BG is composed of at least six different compounds. The weight loss of degradation step I happens between 120 °C and 180 °C, showing a maximum degradation rate at 160 °C and a weight loss of 7.6%. This relates to structural or chemical water molecules belonging to the BG network that were not completely expelled during drying. Step number II starts at 180 °C; it is probably related to the dissociation of O-acetyl side-chain fragmentation reactions to form acetic acid, and a second fragmentation reaction to form some light volatiles [66]. It reaches a maximum degradation rate at 222 °C, and ends at 242 °C, when step number III starts. The latter induces a weight loss of 34.3% and it ends at 322 °C after reaching a maximum degradation rate at 298 °C. It is attributed to the depolymerization reaction of the xylopyranose chain of the hemicellulose [66]. A slope change can be noticed at 322 °C, which marks the beginning of degradation step number IV, with a maximum rate at 347 °C, due to cellulose degradation [66] corresponding to a 14.8% of weight loss. Then, at 383 °C the slope changes again, marking the linear step, V, with a constant degradation rate, which ends at 570 °C, resulting in a weight loss of 12.7%. Over this temperature, weight loss at step number VI, with a very fast degradation, is observed when O_2_ atmosphere is introduced, and it finishes at 582 °C, leading to a weight loss of 16.4%. The degradation is completed at 650 °C, and, above this temperature, the solid residue is about 3 wt. %. TGA and DTG results are summarized in Table 8.

From XRD analysis (Figure 1c), it has been shown the BG powder is mainly amorphous. However, it has ordered crystalline fractions, as suggested by XRD spectra. The main peak occurs at a 2-theta angle of 21.26°, and corresponds to a *d-spacing* of 4.18 Å. It can be attributed to the crystalline fraction of cellulose [67].

### 3.2. Pellets and Filament Thermal Properties

Before printing, the properties of both produced pellets and filament composites were analyzed, starting with the thermal behavior.

**Figure 2:**
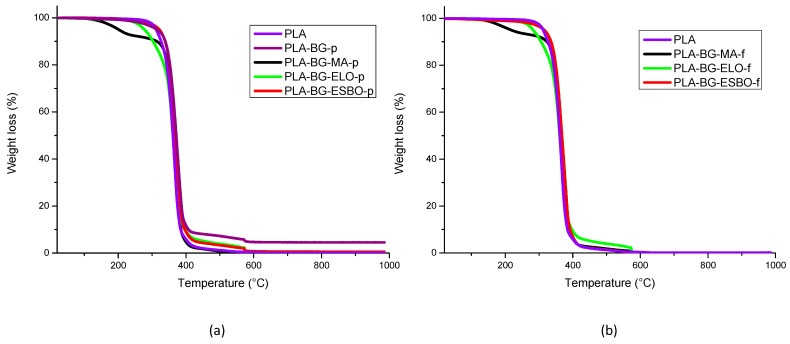
TGA curves of formulations in pellet (**a**) and formulations in filament (**b**).

Considering TGA results for both pellets and filaments, reported in Figure 2a,b, it can be observed that the thermal stability of PLA is affected by the presence of both BG and compatibilizer.

The starting and ending degradation temperatures, the maximum rate degradation temperature (T_dm_), and the weight loss of each degradation step, measured starting from the curves of Figure 2, are reported in Table 9. For the PLA–BG–MA formulation, it can be seen a first degradation step related to the MA’s evaporation happens at 200 °C [68]. PLA–BG–ELO shows an initial degradation temperature lower than that of neat PLA, because ELO starts to degrade at 250 °C and then its degradation is superimposed on that of PLA [48,49]. However, ESBO has a degradation range that completely overlaps with that of PLA [48]. Therefore, all formulations exhibit similar behavior to PLA, degrading almost completely between 300 °C and 400 °C, but MA is less thermally stable than the other two additives. In fact, the degradation of PLA–BG–MA starts at lower temperatures (125 °C), which makes the PLA–BG–ELO and PLA–BG–ESBO formulations more suitable for printing temperatures and possible subsequent processing steps.

After this main step, in some cases, a final section of the curve relating to the degradation of certain BG components can be seen.

**Figure 3:**
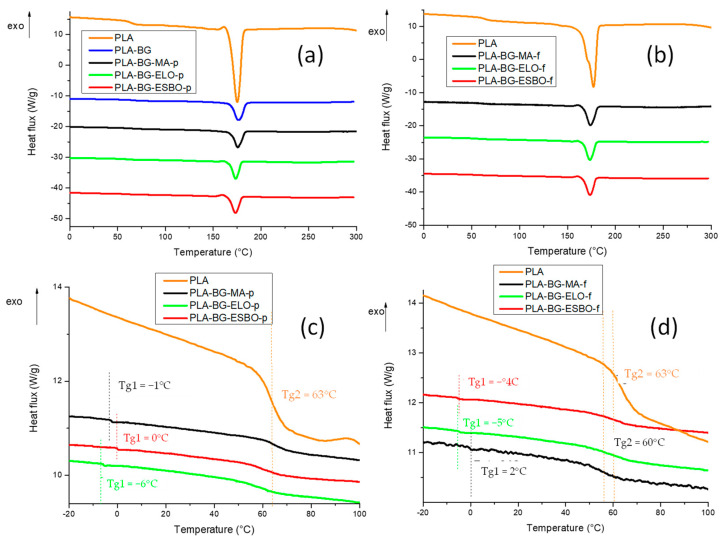
DSC test of formulations in pellet (**a**) and formulations in filament (**b**). Zoom on Tg of the DSC test of formulations in pellet (**c**) and formulations in filament (**d**). The heat flux is exothermic upwards.

Changes in thermal properties with respect to virgin PLA were investigated, measuring the glass transition temperature (Tg) and melting temperature (Tm) of all formulations by using DSC. The thermograms obtained from the DSC analysis are shown in Figure 3a,b, while in Figure 3c,d the zoom on glass transition temperature of PLA and plasticized PLA is reported. 

As can be observed, pristine PLA shows, both for pellet and filament, a single glass transition at 63 °C. However, two glass transitions are visible for PLA plasticized with the three compatibilizers. This indicates that although the plasticization process occurs with the addition of each compatibilizer, some phase segregation happens during processing, due to an inhomogeneous mixing of the plasticizers into PLA. Therefore, two domains can be detected in plasticized PLA: the first one, characterized by lower glass transition temperatures (about 0 °C) with a higher content of plasticizer, and the second one, at higher glass transition temperatures (about 60 °C), rich in PLA content, with a very low amount of plasticizer.

The values of the characteristic temperatures are also summarized in Table 10.

MFI measurements were also carried out on pellets and filaments. The results obtained are reported in Table 11. For the PLA–BG–MA-f sample, it was not possible to measure MFI because of its low viscosity. As can be seen from the MFI values of Table 11, the introduction of MA induced an improvement in the processability of the composite, due to the reduction in viscosity, but in filament form, the viscosity has been significantly reduced. This would make processing less smooth, as the viscosity of the fluid must be in a range suitable for the temperatures employed. Otherwise, the formulations with ELO or ESBO show a lower MFI, especially for the pellet-shaped ones, which means more difficult processability, but the viscosity is still adequate for the processes to which the various formulations were subjected. Despite this, no particular difficulties were encountered at the processing stage.

### 3.3. Thermal Properties of 3D-Printed Specimens

To appreciate eventual changes in thermal properties, the same characterizations performed on feed printing materials (in pellets and filaments) were repeated on the tensile specimens printed by the two different 3D printers. No changes in glass transition and melting temperature were observed from DSC tests on 3D-printed samples compared to pellets and filament composites. Moreover, TGA results show, again, no important variations in thermal stability and degradation temperatures of the 3D-printed samples, as can be seen in Figure S1, reported in the supplementary information section.

### 3.4. Mechanical Properties of 3D-Printed Specimens

**Figure 4:**
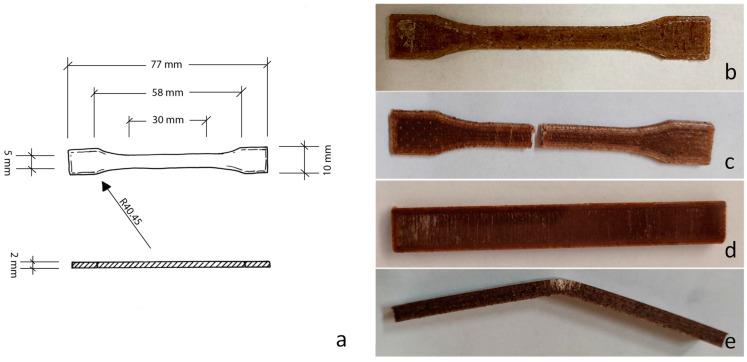
Dimension (in mm) of a tensile specimen (**a**) of PLA–BG–ELO-f before tensile test (**b**) and after tensile test (**c**); rectangular specimen before bending test (**d**) and after bending test (**e**).

The 3D-printed tensile specimens were tested from a mechanical point of view. An example of a tested sample is reported in Figure 4, where the dimensions of the specimen are also indicated, in mm. Firstly, tensile tests were carried out, and the results are shown in Table 9 and Table 10, for the sample printed using Tumaker NX Pro Pellets (labelled with “-Tum”) and by BQ Witbox 2 (labelled with “-Wit”), respectively. Analogously, Table 11 and Table 12 report the bending behaviour of the tested samples, and Table 13 and Table 14 show impact strength. These last two tests were carried out with rectangular specimens (Figure 4d,e).

The introduction of BG to PLA had a stiffening and embrittlement effect, as shown by the Young’s modulus increase, and strength and elongation reduction. However, when adding the compatibilizer, the stiffness decreases, as well as tensile strength (**σ_t_**) and elongation at break (**ε_t_**), as demonstrated by the values reported in Table 12 and Table 13. However, comparing the properties of the specimens realized by the printer Tumaker NX Pro Pellets and by the BQ Witbox 2, the former allowed for obtaining a reduction in a less important properties, probably due to fewer steps of processing of the raw material introduced in the printer. In fact, in the first case, the Young’s modulus (**E_t_**) drops 7–21% based on the compatibilizer), and in the second, the decrease observed is 24–26%. Regarding **σ_t_**, the samples realized by Tumaker NX Pro Pellets show a reduction due to the addition of the compatibilizer of 29–31%, but **ε_t_** is reduced in the PLA–BG formulation but increased to exceed that of PLA due to the presence of the compatibilizer. Employing the other printing technique, they shows a reduction between 38% and 42%, and of 28%, respectively. The three different compatibilizers act in a very similar way, reporting an analogous response in terms of reduction in PLA properties. 

From bending tests, a non-important reduction in stiffness is observed in the different formulations, regardless of printing system, as can be seen in Table 14 and Table 15. Considering samples realized through Tumaker NX Pro Pellets, the maximum bending strength of pure PLA of 78 MPa was reduced when adding BG by 19% and subsequently when adding MA, ELO, or ESBO by 42%, 32%, and 33%, respectively (Table 14). With regard to samples made with BQ Witbox 2, the bending strength suffered a reduction from 81 MPa of the pure PLA specimen of 34% by adding MA and 39% by adding ELO or ESBO (Table 15). This behaviour demonstrates a better suitability of Tumaker NX Pro Pellets in the case of PLA–BG–ELO and PLA–BG–ESBO formulations, while for PLA–BG–MA the BQ Witbox 2 was more appropriate due to a lower loss of properties. At the same time, a reduction in elongation at break from 10% of pure PLA to 7% was observed with the introduction of BG in the PLA matrix, but the addition of the compatibilizer allowed for a new increase up to 11–12% in the case of ELO and ESBO, regardless of printing system.

The mechanical behavior was also investigated through impact tests, the results of which are given in Table 16 and Table 17. In general, the impact strength of samples realized by Tumaker NX Pro Pellets was the same as that of samples made with BQ Witbox 2. Specifically, in the former case, the introduction of bagasse alone led to a 60% reduction in impact strength compared to a pure PLA sample, probably due to poor adhesion between the polymer chains and BG granules. With the addition of the compatibilizer, the loss of strength became 48%, 46%, and 48%, with reference to MA, ELO, and ESBO, respectively. These additives, therefore, resulted in an improvement in the polymer–BG interface. In the latter case, a lower loss of impact strength is found, although the impact strength shown by a pure PLA sample printed with BQ Witbox 2 is lower than that of the same sample printed with Tumaker NX Pro Pellets. In fact, the reduction observed for the three formulations was 40%, 37%, and 47%. However, the final properties achieved, in terms of impact resistance, are the same. Therefore, regardless of the printing technique, the PLA–BG–ELO formulation suffers the least reduction in impact strength compared to pure PLA, so ELO seems to show better compatibilization properties than the other additives.

In general, ELO and ESBO would seem to be more suitable for PLA–BG compatibility, since they are more thermally stable than MA, and result in acceptable mechanical properties.

**Figure 5:**
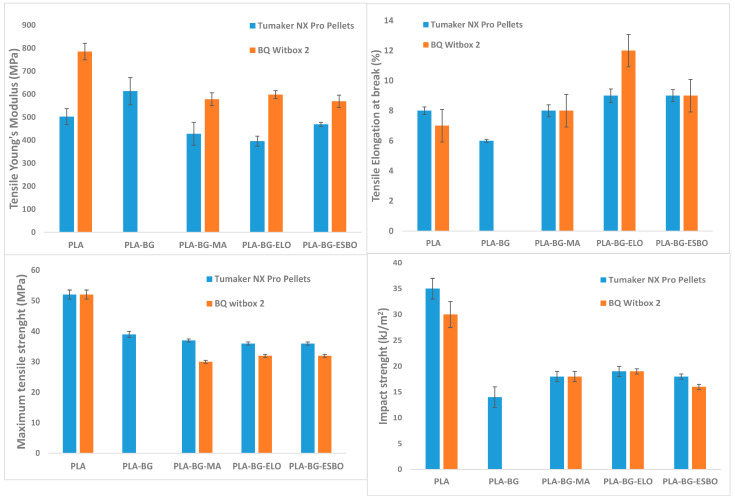
Comparison of mechanical properties of sample realized by Tumaker NX Pro Pellets and by BQ Witbox 2.

A comparison of the different mechanical properties is reported in Figure 5. Observing the behavior of the three compatibilizers, it is possible to see how the effect imparted on the mechanical properties is almost the same for all three. They are all capable of promoting better dispersion of the filler in the PLA matrix in a very similar way, thanks to the good compatibility with PLA. Of course, a drop in mechanical properties due to the presence of a waste was expected. However, this reduction in mechanical properties is not such as to prevent the developed formulations from finding an application. In addition, the need to add a compatibilizer improves the polymer–BG interface, making the printed objects more cohesive and improving the quality of the printing, but on the other hand, it causes an increase in ductility. Therefore, considering the entity of the reduction in properties, we can consider the developed formulations suitable for some applications where high requirements are not necessary, thus, meaning the beer bagasse can be used profitably.

Results of statistical analysis ANOVA are reported in the following tables (Table 18, Table 19, Table 20, Table 21, Table 22 and Table 23). Considering the kind of plasticizer as the source of variation, with three levels, and two degrees of freedom, its significance on tensile modulus, flexural modulus, and impact resistance for samples obtained both from filament and pellet was tested, and strength was tested by calculating the F value as the ratio of the variance between the means to the variance of the experimental error. The F value was then used in order to calculate the corresponding *p*-value, which was then compared with the confidence level, α = 0.05. According to ANOVA, *p* > α corresponds to the null hypothesis (equivalence of the means), whereas *p* < α indicates that the population means were significantly different. For tensile modulus, *p* = 0.56947 and *p* = 0.19675 for samples produced from filament and pellet, respectively, indicating that the corresponding mechanical properties were not influenced by the change of the plasticizer in the tested range of compositions. The same result was obtained for the flexural modulus of samples obtained from filament: with a *p* = 0.83922, flexural strength is not statistically affected by the type of plasticizer.

In contrast, *p* = 0.01064 for the flexural modulus of samples obtained by pellets indicates the statistically relevant effect of the addition of different plasticizers on the corresponding property of the blend. 

Finally, for impact resistance, *p* = 0.0522 and *p* = 0.3421 were found for samples produced by filament and pellets, respectively, thus, indicating that the addition of different plasticizers was not statistically significant. 

### 3.5. Optical Microscopy

**Figure 6:**
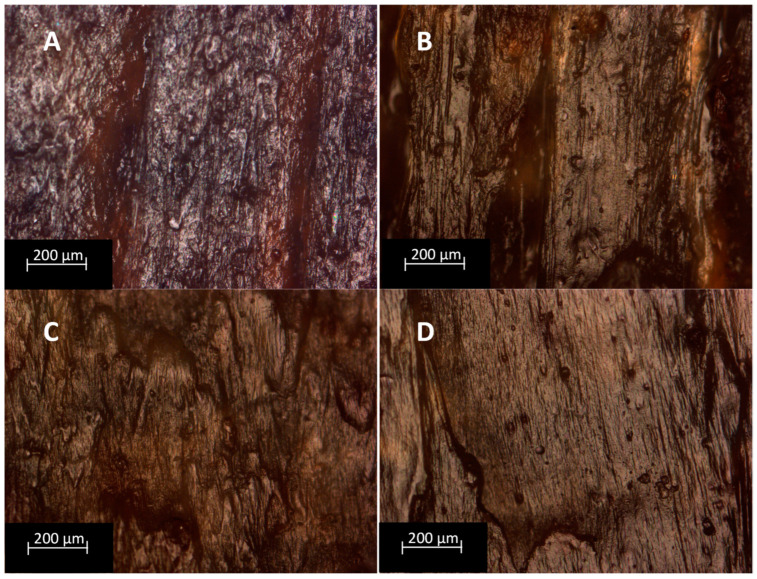
Optical microscope photographs with a magnification of 10× of formulations printed by Tumaker NX Pro Pellets: (**A**) PLA–BG, (**B**) PLA–BG–MA-Tum, (**C**) PLA–BG–ELO-Tum, (**D**) PLA–BG–ESBO-Tum.

**Figure 7:**
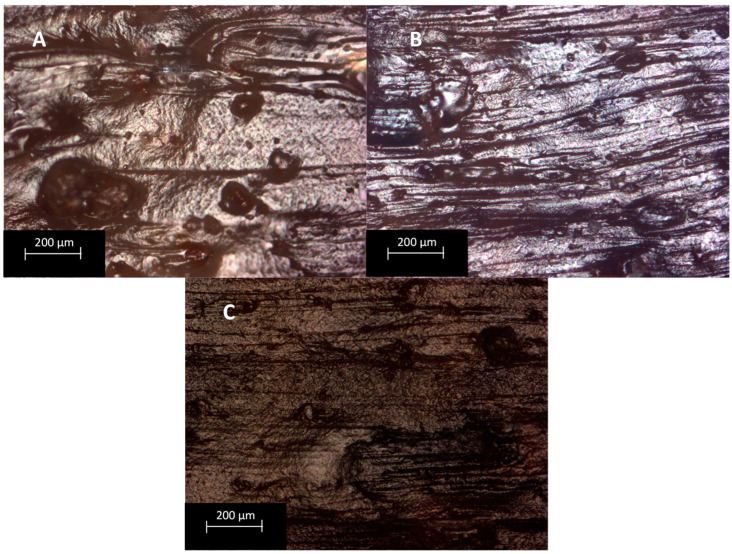
Optical microscope photographs with magnification of 10× of formulations printed by BQ Witbox 2: (**A**) PLA–BG–MA-Wit, (**B**) PLA–BG–ELO-Wit, (**C**) PLA–BG–ESBO-Wit.

The quality of 3D-printed models by using the two different printing techniques was assessed by observing the printed samples under an optical microscope. Considering the specimens printed with the Tumaker NX Pro Pellets printer, shown in Figure 6, it can be seen that the absence of compatibilizer results in a very rough surface (Figure 6A). The introduction of a compatibilizer instead results in more homogenous surfaces with better definitions (Figure 6B–D). When comparing these samples with those printed with the BQ Witbox printer (Figure 7), it can be seen that the latter technique results in less homogenous and defined specimens. In some cases, it is also possible to appreciate the presence of a higher number of larger voids. In particular, the formulation containing MA has large voids, of about 200 µm. Therefore, considering the quality of the printed samples, it can be said that it decreases with increasing the number of processing steps the mixtures undergo.

**Figure 8:**
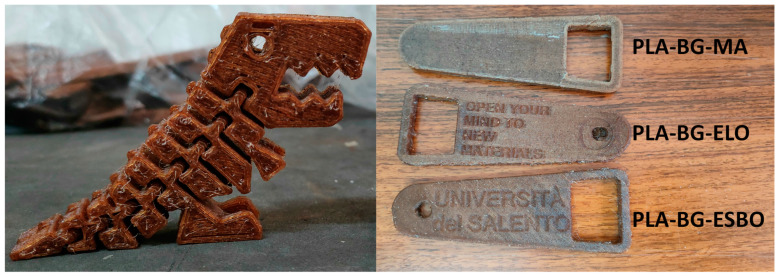
Representative objects printed by Tumaker NX Pro Pellets: on the left, an object manufactured using PLA–BG–ELO formulation; on the right, three objects manufactured using PLA–BG–MA, PLA–BG–ELO, and PLA–BG–ESBO formulations, top down, respectively.

All the scientific results obtained from this study confirm the stabilizing effect of each compatibilizer, evidencing that the Tumaker NX Pro Pellets printer is able to minimize the number of defects of the printed models, allowing the printability of less basic shapes with higher precision, as demonstrated by the representative sample printed by Tumaker NX Pro Pellets, using PLA–BG–MA, PLA–BG–ELO, and PLA–BG–ESBO pellets (Figure 8).

## 4. Conclusions

The present work shows a profitable and efficient possibility in the use of a waste that is currently not valorized. Beer bagasse was, in fact, found to be a possible filler to be used in the preparation of bio-composites, which would be able to reduce the quantity of virgin polymer necessary for the production of feed materials for AM. PLA-based bio-composites in forms of pellets and filaments suitable for MEX 3D printing process were developed, by recovering a waste material of the beer production chain. The effect of different kind of compatibilizer (MA, ELO, and ESBO) on the physical–chemical properties of the bio-composites was assessed. Finally, two different extrusion-based 3D printing techniques were used to evaluate the printability of the developed bio-composites. The following are the relevant conclusions that can be drawn from this work:

- Among the various additives used, MA was the one that made processing convenient thanks to the low viscosity shown at working temperatures. However, at the same time, it was not very thermally stable, since its degradation starts at 125 °C;
- On the other hand, PLA–BG–ELO and PLA–BG–ESBO show very similar mechanical properties, but ELO would seem to be better suited to preserve impact strength properties, probably due to a better polymer–BG interface that it is able to create;
- It was observed that the addition of BG and compatibilizer led to a reduction in stiffness, but the presence of the compatibilizer was useful to improve elongation at break and impact strength with respect to the formulation without the compatibilizer, thanks to a better compounding of the elements and a better cohesion;
- In general, a loss of mechanical properties with respect to pure PLA samples was observed, but the characteristics are still high enough to be used in several applications allowing for the recovering of a waste;
- The 3D samples printed by Tumaker NX Pro Pellets show a higher tensile strength than those made by BQ Witbox 2, and also a better accuracy in printing. It can, hence, be concluded that the extrusion of pellets produced objects with a better quality than those produced through filament extrusion, probably due to the fewer steps of processing necessary for the production of the pellets.

## Figures and Tables

**Table 1:** Main properties of PLA 3D870, according to the technical datasheet [30].

Properties	PLA 3D870	ASTM Method	Ref.
Specific gravity, g/cc	1.22	D792	[31]
MFI, g/10 min	9–15	D1238	[32]
Peak melt temperature, °C	165–180	D3418	[33]
Glass transition temperature, °C	55–60	D3418	[33]
Heat distortion temperature, °C	75–85	E2092	[34]

**Table 2:** Main properties of MA, according to the technical datasheet [35].

Properties	Values
Density at 55 °C	1.32 g/cm^3^
Appearance	Flakes, white, up to 0.5 cm
Bulk density	700–800 kg/m^3^
Solubility in water at 20 °C	Hydrolysis reaction
Melting point	51–53 °C
Boiling point	200 °C
Flash point	103 °C
Ignition temperature	475 °C
Vapor pressure at 40 °C	1.3 hPa
Evaporation heat at 54.9 °C	157.2 KJ/mol
Expl. limit (upper)	7.1 Vol. %
Expl. limit (lower)	1.4 Vol. %
pH	<1

**Table 3:** Main properties of ELO, according to the technical datasheet [36].

Properties	Values
Density at 20 °C	1.05–1.06 g cm^−3^
Viscosity at 25 °C	8–12 p
Acid value	≤1 mg KOH g^−1^
Partition coefficient	>6.2
Iodine value	≤5
Color	Yellowish
Flash point	Non-measurable
Pour point	−3 °C
Oxirane oxygen	>8 °C
Solubility in water	Insoluble
Appearance	Viscous

**Table 4:** Main properties of ESBO, according to the technical datasheet [37].

Properties	Values
Density at 20 °C	0.990–0.997 g cm^−3^
Viscosity at 20 °C	4.5–5.5 cp
Acidity	≤0.75 mg KOH g^−1^
Refractive index at 20 °C	1.472–1.473
Iodine value	≤3
Gardner color	≤2
Flash point	289 °C
Pour point	<12 °C
Oxirane oxygen	6.6–8.0%
Saponification index	182–184
Solubility in water	Insoluble
Appearance	Clear liquid

**Table 5:** Composition of the formulations in the form of pellets.

Formulation	PLA (wt. %)	BG (wt. %)	Compatibilizer (wt. %)
PLA	100	0	0
PLA–BG-p	90	10	0
PLA–BG–MA-p	85.71	9.53	4.76
PLA-BG–ELO-p	85.71	9.53	4.76
PLA–BG–ESBO-p	85.71	9.53	4.76

**Table 6:** Processing parameters of the filament extrusion for each formulation.

Formulation	Temperature Profile (°C)	Screw Speed (rpm)
PLA	170-185-180-180	3.4
PLA–BG–MA-f	170-185-190-170	3.5
PLA–BG–ELO-f	170-185-190-170	3.5
PLA–BG–ESBO-f	170-175-170-160	3.3

**Table 7:** The 3D printing parameters used for the production of tensile, flexure, and impact specimens.

Parameters	BQ Witbox 2	Tumaker NX Pro Pellet
Nozzle diameter (mm)	0.8	0.8
Layer thickness (mm)	0.4	0.4
Extrusion multiplier	1.1	7.5 ^1^/5.6 ^2^
Shrink rate (mm/s)	30	40
First layer width (%)	90	90
Speed reduction factor first layer	0.5	0.6
Infill (%)	100	100
Printing temperature first layer (°C)	195	195
Printing temperature other layers (°C)	190	190
Bed temperature (°C)	50	60
First layer cooling (%)	0	0
Cooling other layers (%)	100	100

^1^ Tensile specimen; ^2^ flexure and impact specimens.

**Table 8:** TGA and DTG results.

Degradation Step	Temperature Range (°C)	Maximum Degradation Rate (°C)	Weight Loss (wt. %)
I	120-180	160	7.6
II	180-242	222	11.2
III	242-322	298	34.3
IV	322-383	347	14.8
V	383-570	Constant rate	12.7
VI	570-582	579	16.4

**Table 9:** Thermal properties from TGA test of pellets and filaments.

Formulation	Degradation Step (°C)	T_dm_ (°C)	Weight Loss (%)	Solid Residue (%)
PLA-p	270–440	367	98	0
PLA-f	270–440	367	98	0
PLA–BG-p	250–440	375	92	
	570–585	575	4	4
PLA–BG–MA-p	125–250	200	8	
	290–430	375	90	0
PLA–BG–MA-f	125–250	200	7	
	290–430	375	90	0
PLA–BG–ELO-p	240–440	363	95	
	570–585	575	5	0
PLA–BG–ELO-f	240–440	363	95	
	570–585	575	5	0
PLA–BG–ESBO-p	270–450	373	96	
	570–585	575	4	0
PLA–BG–ESBO-f	270–450	370	98	0

**Table 10:** Thermal properties from DSC test of pellets and filaments.

Formulation	Tg1 (°C)	Tg2 (°C)	Tm (°C)
PLA-p	-	63	175
PLA-f	-	63	177
PLA-BG-p	-	65	176
PLA–BG–MA-p	−3	64	176
PLA–BG–MA-f	2	60	174
PLA–BG–ELO-p	−6	62	174
PLA–BG–ELO-f	−5	57	173
PLA–BG–ESBO-p	0	64	173
PLA–BG–ESBO-f	−4	64	173

**Table 11:** MFI values of the formulations in pellets and filaments.

Formulation	MFI (g/10min)
PLA	9–15 ^1^
PLA–BG–MA-p	80.7 ± 18.1
PLA-BG-MA-f	ND
PLA–BG–ELO-p	23.8 ± 2.5
PLA–BG–ELO-f	34.6 ± 3.1
PLA–BG–ESBO-p	21.8 ± 1.9
PLA–BG–ESBO-f	34.2 ± 4.3

^1^ From PLA 3D870 technical datasheet.

**Table 12:** Mechanical properties from tensile tests of sample printed using Tumaker NX Pro Pellets.

Formulation	Young’s Modulus E_t_ (MPa)	Maximum Strength σ_t_ (MPa)	Elongation at Break ε_t_ (%)
PLA	502 ± 70	52 ± 3	8 ± 0.5
PLA–BG-Tum	613 ± 118	39 ± 2	6 ± 0.2
PLA–BG–MA-Tum	428 ± 100	37 ± 1	8 ± 0.8
PLA–BG–ELO-Tum	396 ± 43	36 ± 1	9 ± 0.9
PLA–BG–ESBO-Tum	469 ± 18	36 ± 1	9 ± 0.8

**Table 13:** Mechanical properties from tensile tests of sample printed using BQ Witbox 2.

Formulation	Young’s Modulus E_t_ (MPa)	Maximum Strength σ_t_ (MPa)	Elongation at Break ε_t_ (%)
PLA	785 ± 72	52 ± 3	7 ± 0.6
PLA–BG–MA-Wit	578 ± 56	30 ± 1	8 ± 1
PLA–BG–ELO-Wit	598 ± 34	32 ± 1	12 ± 1.1
PLA–BG–ESBO-Wit	569 ± 53	32 ± 1	9 ± 1

**Table 14:** Mechanical properties from bending tests of sample printed using Tumaker NX Pro Pellets.

Formulation	Young’s Modulus E_f_ (MPa)	Maximum Strength σ_f_ (MPa)	Elongation at Break ε_f_ (%)
PLA	2680 ± 78	78 ± 1	10 ± 0.1
PLA–BG-Tum	2740 ± 411	63 ± 3	7 ± 1.2
PLA–BG–MA-Tum	1940 ± 197	45 ± 2	8 ± 1
PLA–BG–ELO-Tum	2520 ± 51	53 ± 1	11 ± 0.9
PLA–BG–ESBO-Tum	2250 ± 117	52 ± 1	11 ± 1.1

**Table 15:** Mechanical properties from bending tests of sample printed by BQ Witbox 2.

Formulation	Young’s Modulus E_f_ (MPa)	Maximum Strength σ_f_ (MPa)	Elongation at Break ε_f_ (%)
PLA	2590 ± 308	81 ± 6	10 ± 0.1
PLA–BG–MA-Wit	2370 ± 78	52 ± 3	8 ± 0.8
PLA–BG–ELO-Wit	2320 ± 39	49 ± 2	12 ± 0.5
PLA–BG–ESBO-Wit	2290 ± 96	49 ± 2	12 ± 0.2

**Table 16:** Mechanical properties from impact tests of sample printed using Tumaker NX Pro Pellets.

Formulation	Impact Strength (kJ/m^2^)
PLA	35 ± 4
PLA–BG-Tum	14 ± 4
PLA–BG–MA-Tum	18 ± 2
PLA–BG–ELO-Tum	19 ± 2
PLA–BG–ESBO-Tum	18 ± 1

**Table 17:** Mechanical properties from impact tests of sample printed using BQ Witbox 2.

Formulation	Impact Strength (kJ/m^2^)
PLA	30 ± 5
PLA–BG–MA-Wit	18 ± 2
PLA–BG–ELO-Wit	19 ± 1
PLA–BG–ESBO-Wit	16 ± 1

**Table 18:** ANOVA analysis: tensile modulus of samples made from filament.

	Degree of Freedom	Sum of Squares	Mean Square	F-Value	Prob > F
Model	2	5541.33333	2770.66667	0.58472	0.56947
Error	15	71,077.16667	4738.47778		
Total	17	76,618.5			

At the 0.05 level, the population means are not significantly different.

**Table 19:** ANOVA analysis: tensile modulus of samples made from pellets.

	Degree of Freedom	Sum of Squares	Mean Square	F-Value	Prob > F
Model	2	34,064.33333	17,032.16667	1.81551	0.19675
Error	15	140,722.16667	9381.47778		
Total	17	174,786.5			

At the 0.05 level, the population means are not significantly different.

**Table 20:** ANOVA analysis: flexural modulus of samples made from filament.

	Degree of Freedom	Sum of Squares	Mean Square	F-Value	Prob > F
Model	2	7900	3950	0.17734	0.83922
Error	15	334,100	22,273.33333		
Total	17	342,000			

At the 0.05 level, the population means are not significantly different.

**Table 21:** ANOVA analysis: flexural modulus of samples made from pellets.

	Degree of Freedom	Sum of Squares	Mean Square	F-Value	Prob > F
Model	2	544,533.33333	272,266.66667	6.24544	0.01064
Error	15	653,916.66667	43,594.44444		
Total	17	1,984,500			

At the 0.05 level, the population means are significantly different.

**Table 22:** ANOVA analysis: impact resistance of samples made from filament.

	Degree of Freedom	Sum of Squares	Mean Square	F-Value	Prob > F
Model	2	24.08879	12.0444	3.26334	0.0522
Error	15	110.72442	3.69081		
Total	17	134.81321			

At the 0.05 level, the population means are not significantly different.

**Table 23:** ANOVA analysis: impact resistance of samples made from pellets.

	Degree of Freedom	Sum of Squares	Mean Square	F-Value	Prob > F
Model	2	10.53501	5.26751	1.11063	0.3421
Error	15	147.02731	4.74282		
Total	17	157.56233			

At the 0.05 level, the population means are not significantly different.

## Data Availability

The data presented in this study are available on request from the corresponding author.

## Supplementary Materials

The following supporting information can be downloaded at: https://www.mdpi.com/article/10.3390/polym15214323/s1, Figure S1: TGA curves of PLA–BG–MA in pellet form, filament form, and printed by Tumaker NX Pro Pellets, and by BQ Witbox 2 (a), PLA–BG–ELO in pellet form, filament form, and printed by Tumaker NX Pro Pellets, and by BQ Witbox 2 (b), PLA–BG–ESBO in pellet form, filament form, and printed by Tumaker NX Pro Pellets, and by BQ Witbox 2 (c).
