# Supplementary material for: Poly-Lactic Acid-Bagasse Based Bio-Composite for Additive Manufacturing
Source: Polymers (Basel). 2023 Nov 4;15(21):4323. doi: 10.3390/polym15214323 (PMC10647417; doi:10.3390/polym15214323)
Supplement: Supplementary file 1 [file polymers-15-04323-s001.zip › polymers-2659475-supplementary.pdf]

polymers

Supplementary material

Poly-Lactic Acid–Bagasse-Based Bio-Composite for Additive Manufacturing

Silvia Carichino [1], Dino Scanferla [1], Daniela Fico [1], Daniela Rizzo [2], Francesca Ferrari [1], María Jorda-Reolid [3], Asunción Martínez-García [3] and Carola Esposito Corcione [1,*]

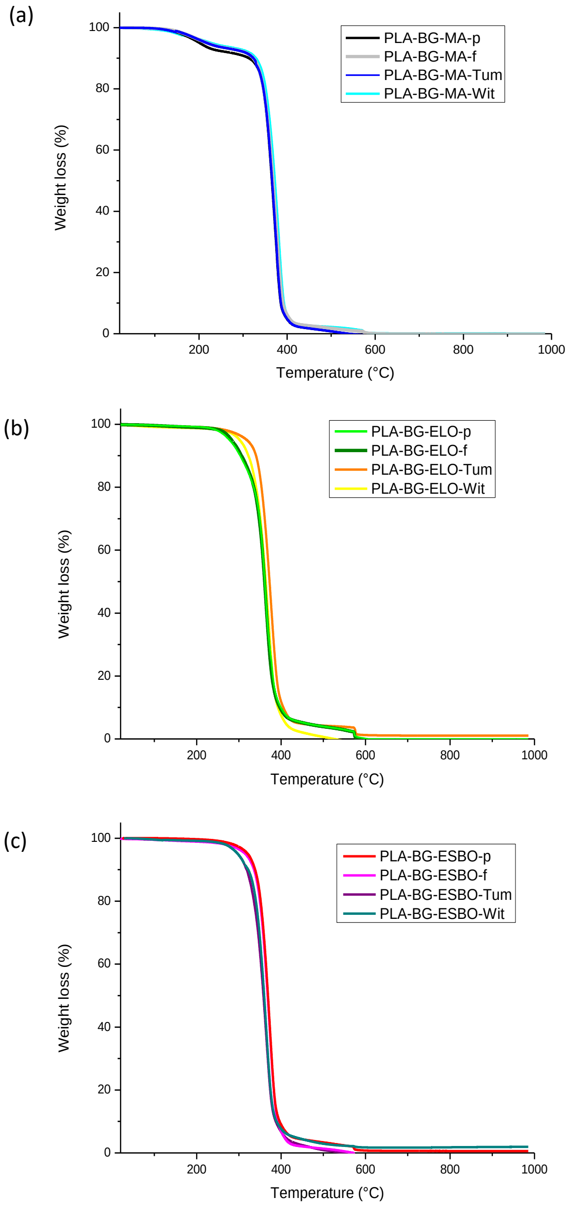

Figure S1. TGA curves of PLA–BG–MA in pellet form, filament form, and printed by Tumaker NX Pro Pellets, and by BQ Witbox 2 (**a**), PLA–BG–ELO in pellet form, filament form, and printed by Tumaker NX Pro Pellets, and by BQ Witbox 2 (**b**), PLA–BG–ESBO in pellet form, filament form, and printed by Tumaker NX Pro Pellets, and by BQ Witbox 2 (**c**).
